# Dental implants after the use of bichat’s buccal fat pad for the sealing of oro-antral communications. A case report and literature review

**DOI:** 10.4317/jced.53318

**Published:** 2016-12-01

**Authors:** Cosimo Galletti, Giovanni Cammaroto, Francesco Galletti, Octavi Camps-Font, Cosme Gay-Escoda, José-Javier Bara-Casaus

**Affiliations:** 1 DDS, MS. Master degree program in Integrated Adult Dentistry. Faculty of Dentistry - University of Barcelona. Department, Hospital Universitari Sagrat Cor. Barcelona, Spain; 2 MD. Department of Otorhinolaryngology. Faculty of Medicine - University of Messina; 3 MD, PhD. Department of Otorhinolaryngology. Faculty of Medicine - University of Messina; 4 DDS, MS. Master degree program in Oral Surgery and Implantology. Associate professor of Oral Surgery and Professor of the Master degree program of Oral Surgery and Implantology. Faculty of Dentistry – University of Barcelona; 5 MD, DDS, MS, PhD. Chairman and Professor of Oral and Maxillofacial Surgery. Faculty of Dentistry – University of Barcelona. Coordinating investigator of the IDIBELL institute. Head of the Oral and Maxillofacial Surgery Department, Teknon Medical Center. Barcelona, Spain; 6 MD, DDS, MS, PhD. Professor of the Master degree program of Integrated Adult Dentistry. Faculty of Dentistry – University of Barcelona. Head of the Oral and Maxillofacial Surgery

## Abstract

Oro-antral communications are frequent complications in oral surgery, and generally occur after molar extractions, maxillary sinus elevations or dental implant procedures. The presence of these defects may increase the morbidity and often need a surgical approach. The present report describes an oro-antral communication in a 52-year-old female who presented a 2 week-course of painless nasal obstruction and rhinorrea after a right maxillary sinus floor elevationwith simultaneous dental implant placement. Based on the anamnesis, clinical examination and a computed tomographyof the paranasal sinuses, a diagnosis of odontogenic rhinosinusitis associated with a 1.5 cm diameter oro-antral communicationwas establishedand its surgical closure using Bichat’s buccal fat pad was planned.After 15 months, the patient was successfully rehabilitated with an implant-supported 3 unit fixed partial denture.

** Key words:**Dental implants, buccal fat pad, oro-antral communications.

## Introduction

An oro-antral communication (OAC) is a pathological condition characterized by the presence of a communication between the oral cavity and the maxillary sinus secondary to loss of the normally separating soft and hard tissues. OACs are relatively frequent complications in oral surgery and generally occur after upper molars and premolars extractions, dental implant related procedures, cystic or tumoral diseases, infections and trauma. Several factors, such as the location of the defect, its cause and size, condition the treatment strategy (1).

The presence of these defects may lead to some sinus complications (i.e. rhino sinusitis) that always require prior medical-surgical treatment (2).

Several surgical techniques, including the use of local (vestibular and/or palatine) or distant flaps (tongue, temporal muscle, or the pediculate flap of Bichat’s buccal fat pad, among others) as well as the use autogenous block grafts or biomaterials have been proposed for the treatment of OACs (2,3).

The use of the buccal fat pad for sealing OACs was firstly described by Egyedi in 1977 (4). Anatomically, the buccal fat pad is an encapsulated, rounded and biconvex, mainly adipose structure with an excellent blood supply from the maxillary, superficial temporal and facial arteries (5). This triple irrigation system is what makes it possible to use this tissue without much risk of necrosis (4). The fat pad is delimited by the masseter muscle and the ascending mandibular ramus and zygomatic arch (6,7).

The aim of the present case report was to describe the main clinical features and treatment of a patient diagnosed with OAC. The study protocol was approved by the Ethical Committee for Clinical Research (CEIC) of the Dental Hospital of the University of Barcelona.

## Material and Methods

A 52-year-old woman came to the Oral Surgery Unit of the Master degree program in Integrated Adult Dentistry of the School of Dentistry of the University of Barcelona presenting a 2 week-course of painless nasal obstruction and rhinorrea after a right maxillary sinus floor elevation thorough a lateral window approach with simultaneous dental implant placement in the position of the right upper first molar (#1.6).

Patient’s pathological background comprised chronic gastroesophageal reflux and depression, treated with metoclopramide 10 mg orally (1 every 24 hours. (Primperan®, Sanofi, Barcelona, Spain))and fluoxetine 20 mg orally (1 every 24 hours. (Fluoxetina Cinfa®, Cinfa, Huarte, Spain)), respectively. Neither allergies nor toxic habits were referred.

The patient had been diagnosed with maxillary rhinosinusitis of odontogenic origin, which was unsuccessfully treated with implant removal and clindamycin 300 mg (1 every 6 hours during the first 7 days. [Dalacin®, Pfizer; Madrid, Spain]), amoxicillin and potassium clavulanate 500/125 (1 every 8 hours during the first 7 days. [Augmentine 500/125®; GlaxoSmithKline, Madrid, Spain]) andamoxicillin and potassium clavulanate 875/125 (1 every 8 hours during the last 7 days. [Augmentine 875/125®; GlaxoSmithKline, Madrid, Spain]).

**Figure 1:**
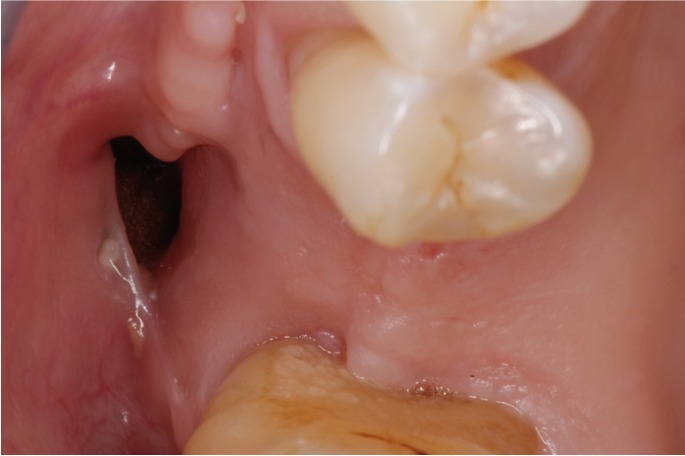
Baseline: clinical features.

Following the anamnesis, a general, regional and local exploration was performed.Rounded painless OAC of 1.5 cm diameter located at the buccal aspect of the right maxillary molar region (Fig. 1).

A computed tomography of the paranasal sinuses was requested as a complementary test. The results, evaluated by a radiologist, revealed an opacification of the right maxillary sinus with multiple radio-opaque masses –compatible with biomaterial particles- and bone resorption of the buccal wall.

Based on the data obtained, a diagnosis of OAC was established and its surgical closure using Bichat’s buccal fat pad was planned. The patient was given full information about the surgical procedure and treatment alternatives and duly signed informed consent form.

**Figure 2:**
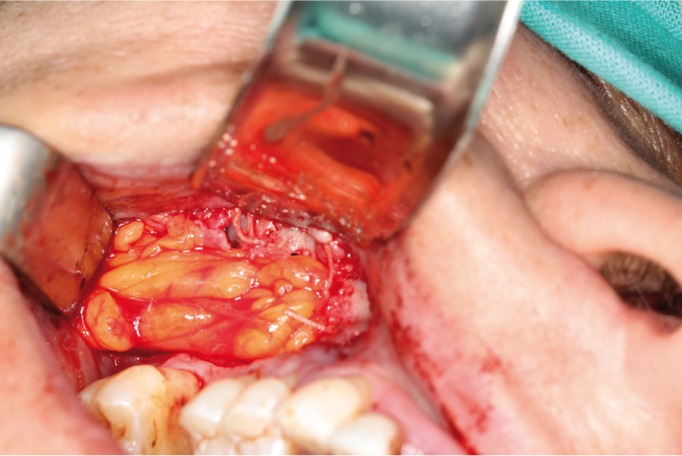
Pedicled graft of Bichat fat pad.

The intervention was performed under local (articaine in a 4% solution of epinephrine 1:100,000 [Artinibsa®; Inibsa Dental, Lliçà de Vall, Spain]) and general anaesthesia. Amucoperiosteal trapezoidal flap was raised extending on each side of the defect and to the bottom of the vestibule. Removal of inflammatory tissue, pus and biomaterial granules was performed under constant irrigation with sterile saline. Then, a 1-cm horizontal incision was made in the reflected periosteum, posterior to the zygomatic buttress. A blunt clamp was introduced towards the temporomandibular angle to localize and prolapse the buccal extension of Bichat´s buccal fat pad in order to cover the OAC entirely. Once placed over the defect, the fat pad was sutured to the surrounding mucosa using 3/0 polyglactin-910 (Ethicon Vicryl®; Johnson & Johnson, New Jersey, United States of America) (Fig. 2) and covered with the mucoperiosteal flap. The mucoperiosteal flap was sutured without tension using 3/0 silk (Aragó, Barcelona, Spain).

After the operation, an antibiotic (500 mg levofloxacin, p.o. every 8 hours for 15 days [Levofluoxacino Ratiopharm; Ratiopharm, Alobendas, Spain]), an analgesic (1 g paracetamol, p.o. every 8 hours for 3-4 days [Gelocatil; Gelos, Barcelona, Spain]), a nasal corticosteroid nasal spray (64 µg/nebulizationbudesonide, every 24 hours for 15 days [Rhinocort®; AstraZeneca, Madrid,Spain]), a sterile saline solution nasal spray (1 nebulization every 12 hours for 15 days and a mouthrinse (15 mL of 0.12% chlorhexidine digluconate every 12 hours for 15 days [Clorhexidina Lacer; Lacer, Barcelona, Spain]) were prescribed. Postoperative instructions and use of prescribed drugs were explained and handed out in a paper sheet that was given to the patient.

**Figure 3:**
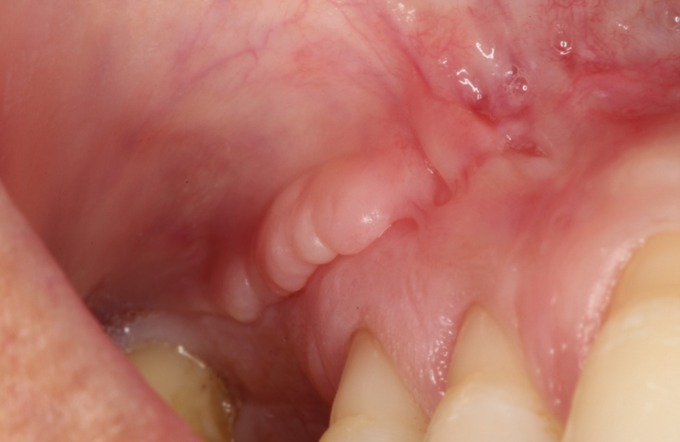
Three-months follow up.

The postoperative period was uneventful and suture was removed after 15 days. At 3 months’ follow-up, the patient did not refer any symptoms and the OAC was completely closed (Fig. 3).

The computed tomography requested twelve months after the surgery revealed no opacification of the right maxillary sinus. Due to periodontal disease, right upper second premolar (#1.5) and second molar (#1.7) were decided to be extracted and replaced with dental implants within 3 months.

A midcrestal incision was made and a full-thickness flap were elevated to expose the alveolar ridge. Implant sites were prepared using drills of increasing diameters, under constant irrigation with sterile saline, according to the manufacturers’ recommendations. Two internal hexagon implants (Ocean HI®, Avinent, Santpedor, España) were placed under local anaesthesia (articaine in a 4% solution of epinephrine 1:100,000 [Artinibsa®; Inibsa Dental, Lliçà de Vall, Spain]) in the position of #15 and #17, preserving the previously treated area. Functional and aesthetic requirements were taken into account to determine the inclination of the implants in mesio-distal and buccolingual directions. The flap was repositioned with 4/0 polyamide suture (Supramid; Aragó, Barcelona, Spain).

After the operation, an antibiotic (750 mg amoxicillin every 8 hours for 7 days orally [Clamoxyl 750®; GlaxoSmithKline, Madrid, Spain]), a nonsteroidal anti-inflammatory drug (600 mg ibuprofen, p.o. every 8 hours for 4-5 days [Algiasdin®; Esteve, Barcelona, Spain]), an analgesic (1 g paracetamol, p.o. every 8 hours for 3-4 days [Gelocatil®; Gelos, Barcelona, Spain]), and a mouthrinse (15 mL of 0.12% chlorhexidine digluconate every 12 hours for 15 days [Clorhexidina Lacer®; Lacer, Barcelona, Spain]) were prescribed. Postoperative instructions and use of prescribed drugs were explained and handed out in a paper sheet that was given to the patient.

The postoperative period was uneventful and suture was removed after 15 days.

No complications were reported during the osteointegration period and the patient was successfully rehabilitated with an implant-supported 3 unit fixed partial denture.

Informed consent for publication was obtained from the patient.

## Discussion

Our paper reports on a case of oral implantation following the reconstruction with BFP in a patient affected by an OAC occurred after a failed right maxillary sinus floor elevation with simultaneous dental implant placement.

Several reports have demonstrated the efficacy of BFP for closing OACs. Moreover, beyond its satisfactory outcomes and feasibility, it is well tolerated by patients (2,3,7,8).

Bichat’s fat consists of a central body and 4 processes: buccal, pterygoid, superficial temporal and deep temporal processes (5). Each process has its own capsule and is anchored to the surrounding structures by ligaments. Due to its rich blood supply, it can be used as a pedicled graft for covering defects in the palatine region or oral mucosa, to seal oronasal fistulas, cover bone graft surfaces, and reconstruct posttraumatic defects in the maxillary region. In particular, the body and buccal process can be easily reached through the oral cavity and used for these purposes (9).

The quick epithelialization of the uncovered fat is a characteristic feature of the pedicled BFP flap (9,10). Hence, a crucial point for its surgical success is the careful and gentle preparation of the BFP since excessive pulling or fragmenting of the tissue might-jeopardizethe vascular supply of the flap and result in a complete failure of the graft (12).

The real function of BFP still remains unclear. Nevertheless, it has been suggested that could prevent fromnegative pressure in newborns while sucking, separate the masticator muscles from one another and from the adjacent bony structures, enhance of intermuscular motion, and protection of the neurovascular bundles (7,8).

A review of the literature from around 500 cases on BFP for closing OACs was performed in order to summarize the main clinical features, complications and prognosis of this technique (2,6,8,9-15) ( Table 1). The most frequent complications described in the literature are infection, necrosis or partial rupture of the flap.In our clinical case, BFP positioning was successful and guaranteed a complete closure of the OAC without any particular complications. Furthermore, the following dental implant procedure on the previously treated area resulted in a satisfactory outcome.

**Table 1:**
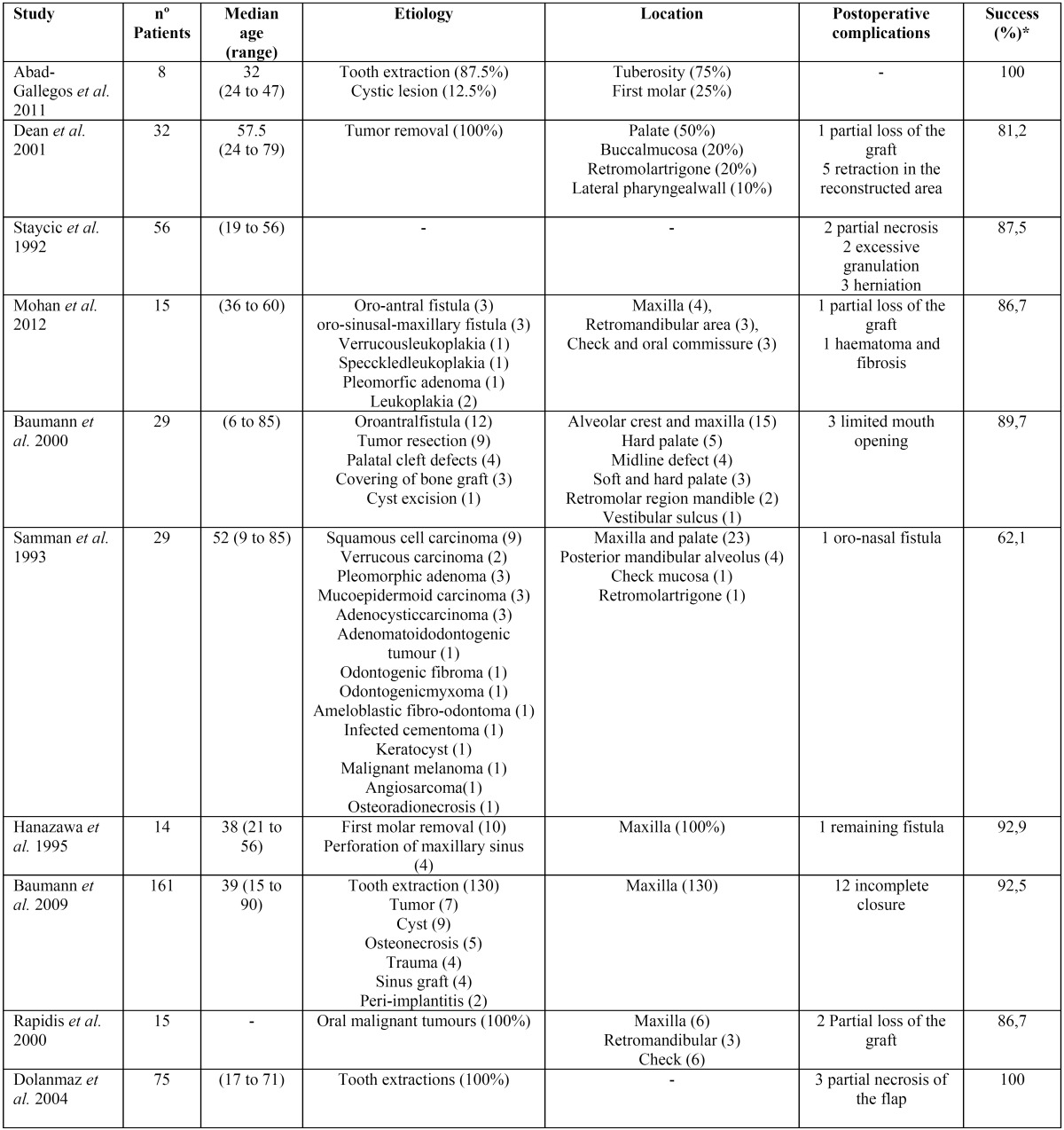
Literature review.

Several other surgical procedures have been described for successful management of OACs. The most widespread technique is the buccal or palatal advancement flap, which probably represents the standard procedure for small OACs closure. However, despite the simplicity and safety of the procedure, it has some drawbacks. Firstly, it results in a decreased vestibular sulcus depth, thus compromising the prosthetic rehabilitation. In addition, it cannot be applied in cases in which the gingival region has been severely damaged. Finally, its success is questionable in challenging cases of previously operated OACs (6,10). On the contrary, when large OACs are needed to be treated or pedicled BFP flap fails to resolve the defect, the use of more aggressive treatments including distant muscular flaps –tongue,temporal, etc.-, autogenous block grafts or the use of biomaterials, are required (2).

In conclusion, the use of a pedicledflap with the buccal fat pad is a simple and rapid technique that can be used to seal defects measuring up to 5 cm in diameter, with no changes in patient anatomy or function- Additional advantages are the great elasticity and excellent blood supply of this anatomical structure, which thus appears as a good treatment option, affording optimum results (2).
